# P-1024. Evaluation of Clostridioides difficile Infection Management among Patients with Polymerase Chain Reaction (PCR) Positive/Toxin Enzyme Immunoassay (EIA) Negative Tests at a Comprehensive Cancer Center

**DOI:** 10.1093/ofid/ofaf695.1220

**Published:** 2026-01-11

**Authors:** Wonhee So, Maryam Jabri, Thu Phu, Justine Abella Ross, Jana Dickter, Rosemary She, Sanjeet S Dadwal

**Affiliations:** Western University of Health Sciences, duarte, CA; Western University of Health Sciences, duarte, CA; Western University of Health Sciences, duarte, CA; City of Hope National Medical Center, Duarte, California; City of Hope National Medical Center, Duarte, California; City of Hope, Duarte, California; City of Hope National Medical Center, Duarte, California

## Abstract

**Background:**

Two-step *Clostridioides difficile* infection (CDI) testing with PCR and EIA has improved diagnostic precision, but clinical significance of PCR+/toxin- (P+T-) is unclear. Our policy recommends against routine treatment (tx) of P+T- patients unless they have signs/symptoms (s/s) of CDI without alternate explanation. In our audit, 49% with P+T- were given CDI tx with 62% ≥2 s/s. This study aimed to assess the conservative estimate of possible PCR+/toxin+ (P+T+) CDI in patients with P+T- result, evaluate factors associated with CDI tx and outcomes including progression to PCR+/toxin+ (P+T+) over 3 months.

**Figure:**
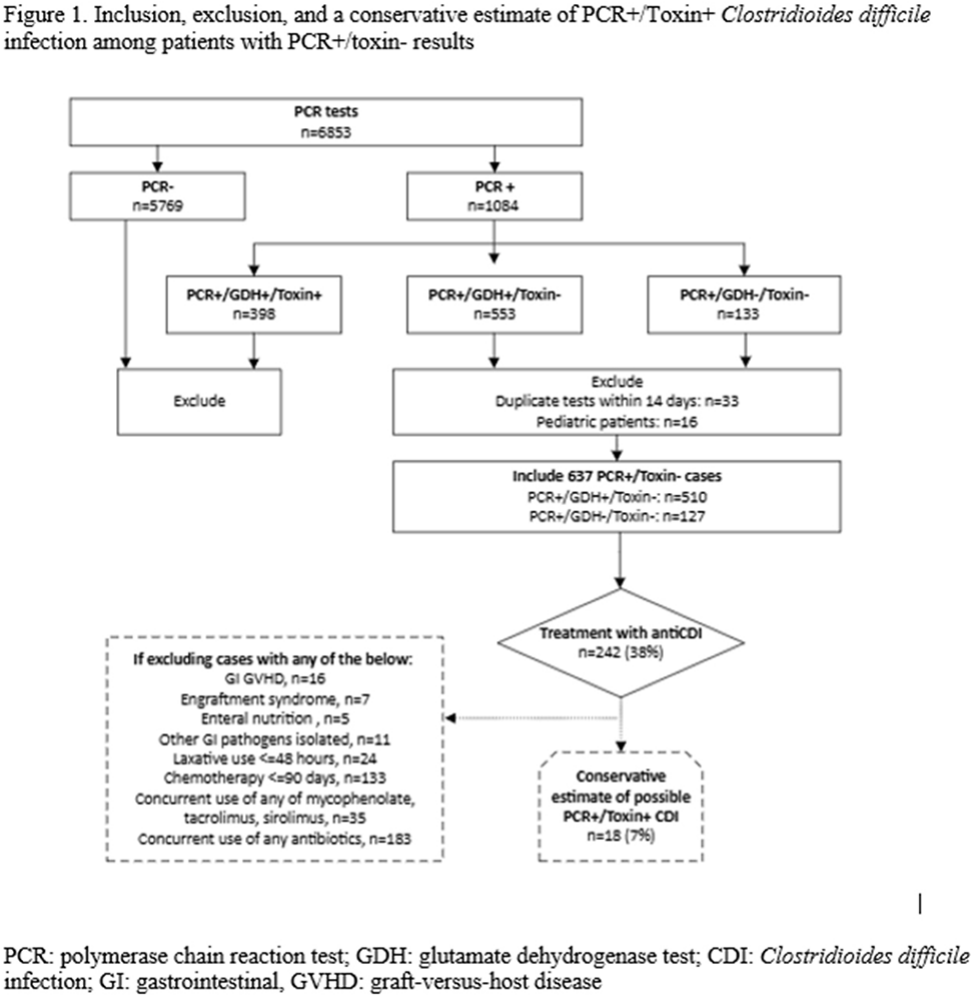

**Figure:**
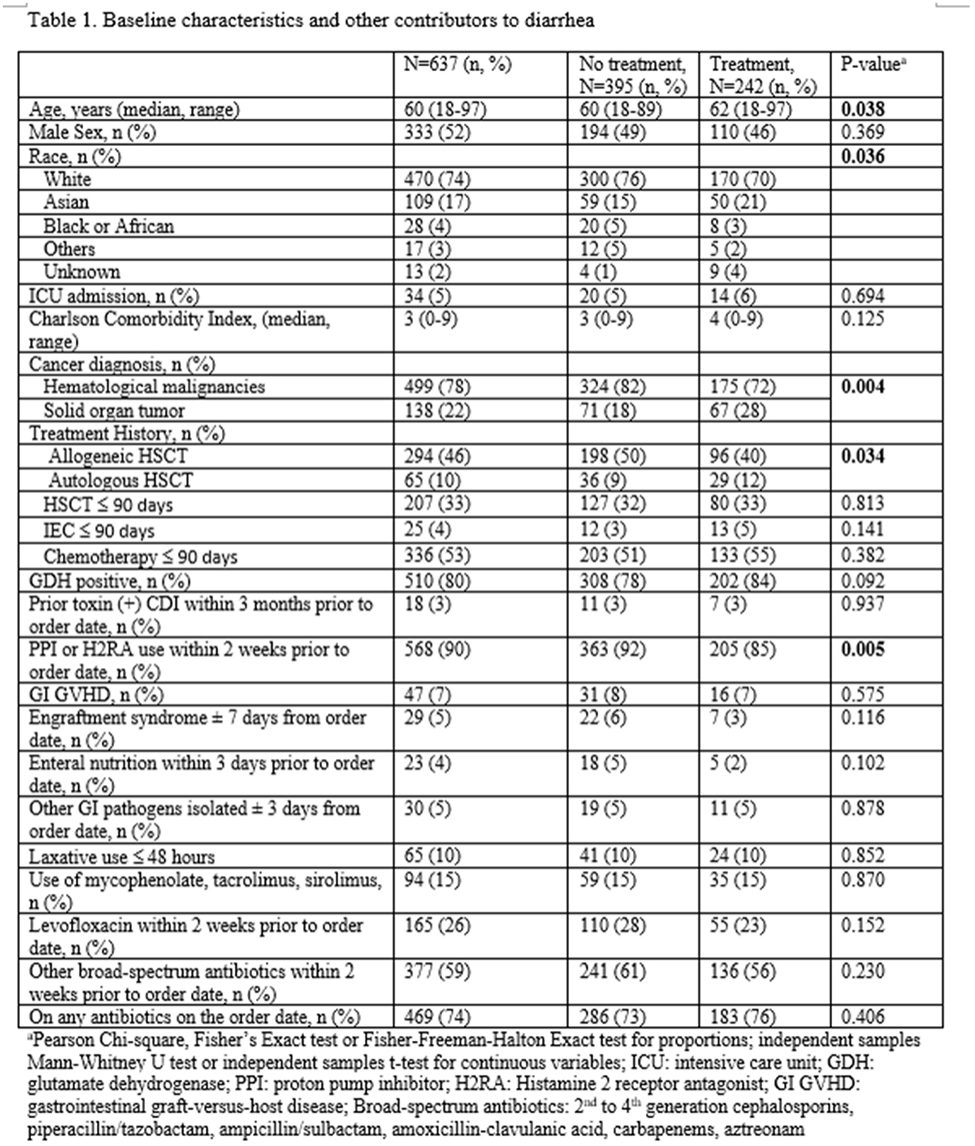

**Methods:**

Retrospective review of adult, hospitalized patients with P+T- from 8/18/2021-8/17/2024 was performed (Figure 1). Duplicate tests ≤14 days were excluded. Pt demographics, potential contributors to diarrhea (Table 1), s/s (Table 2), and clinical and microbiological outcomes based on CDI tx v. no CDI tx were collected (Table 3). CDI tx group was defined as receiving ≥3 days of CDI tx. A conservative estimate of possible P+T+ CDI with P+T- result was determined by counting patients treated with ≥3 days of CDI tx with no other causes of diarrhea. Logistic regression was performed to identify factors associated with starting CDI tx using SPSS v. 29.

**Figure:**
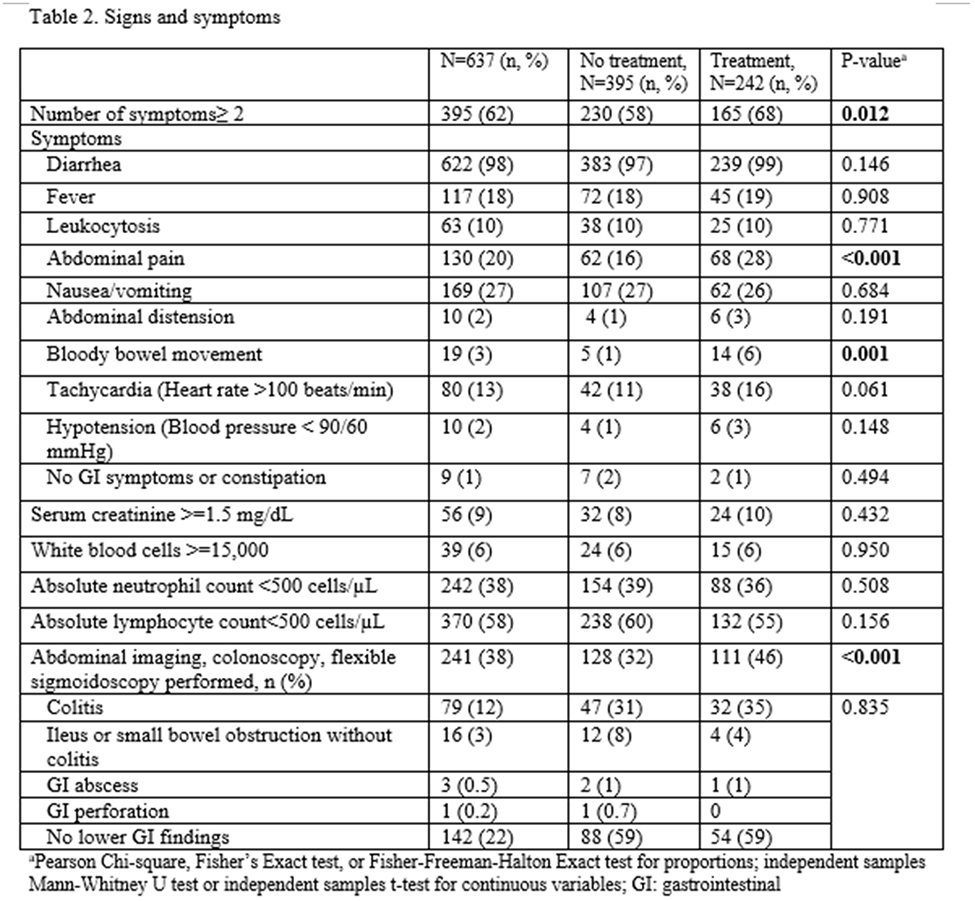

**Figure:**
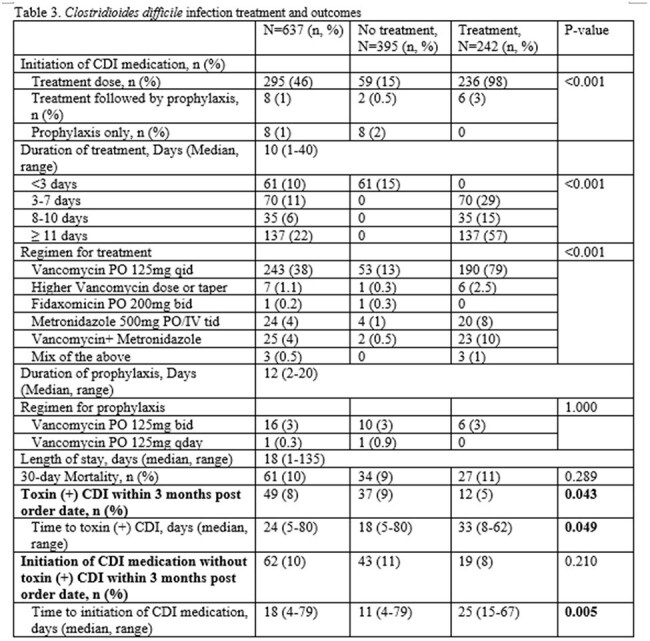

**Results:**

Among 637 P+T– cases: median age 60, male 52%, hematological malignancies 78%, 38% received CDI tx. A conservative estimate of possible P+T+ CDI with P+T- result was 7% (Figure 1, Table 1). Other contributors to diarrhea were similar between groups (Table 1). CDI tx group showed significantly higher rates of ≥2 GI symptoms, abdominal pain, and bloody stool v. no CDI tx (Table 2). CDI tx group had lower rates (5 v. 9%, p< 0.043) of progression to P+T+ CDI with delayed onset (33 v. 18 days, p< 0.049) v. the no CDI tx over 3 months (Table 3). Multivariable regression controlled for immune effector cell therapy and enteral nutrition identified abdominal pain (OR 2.1, p< 0.001) and bloody stool (OR 4.2, p< 0.001) as predictors for starting CDI tx.

**Conclusion:**

CDI tx was driven by clinical presentation over P+/T- result. In this highly vulnerable population, P+/T- untreated group had significantly higher P+/T+ CDI progression over the next 3 months suggesting ongoing selective pressure in colonized patients.

**Disclosures:**

Sanjeet S. Dadwal, MD, Ansun Biopharma: Grant/Research Support|Aseptiscope, Inc.: Stocks/Bonds (Private Company)|Basilea: Advisor/Consultant|Basilea: Grant/Research Support|F2G: Grant/Research Support|Karius: Advisor/Consultant|Karius: Grant/Research Support|Karius: Honoraria|Merck: Advisor/Consultant|Pfizer: Grant/Research Support|Pulmotect: Grant/Research Support|Symbio: Grant/Research Support|Takeda: Advisor/Consultant

